# High-normal levels of hs-CRP predict the development of non-alcoholic fatty liver in healthy men

**DOI:** 10.1371/journal.pone.0172666

**Published:** 2017-02-24

**Authors:** Jieun Lee, Kijung Yoon, Seungho Ryu, Yoosoo Chang, Hyoung-Ryoul Kim

**Affiliations:** 1 Health Promotion Center, Ajou University Hospital, Ajou University School of Medicine, Suwon, Korea; 2 Health Screening Center, Kangbuk Samsung Hospital, Sungkyunkwan University, School of Medicine, Seoul, Korea; 3 Department of Occupational Medicine, Kangbuk Samsung Hospital, Sungkyunkwan University, School of Medicine, Seoul, Korea; 4 Department of Occupational and Environmental Medicine, College of Medicine, the Catholic University of Korea, Seoul, South Korea; Michigan State University, UNITED STATES

## Abstract

We performed a follow-up study to address whether high sensitivity C-reactive protein (hs-CRP) levels within the normal range can predict the development of non-alcoholic fatty liver disease (NAFLD) in healthy male subjects. Among15347 male workers between 30 and 59 years old who received annual health check-ups in 2002, a NAFLD-free cohort of 4,138 was followed through December 2009. Alcohol consumption was assessed with a questionnaire. At each visit, abdominal ultrasonography was performed to identify fatty liver disease. The COX proportional hazard model was used to evaluate the relationship between hs-CRP and incident NAFLD. During the follow-up period, 28.8% (1191 of 4138) of participants developed NAFLD. The hazard ratios of NAFLD were increased by hs-CRP categories within the normal range in the non-adjusted model and age-adjusted model. After adjusting for age, exercise, smoking, BMI, systolic BP, triglyceride, and fasting glucose, these incidences were only increased between the lowest and the highest hs-CRP categories. The risk for NAFLD increased as the hs-CRP level increased (p< 0.001). As the hs-CRP level increased within the healthy cohort, the risk of developing NAFLD increased. This trend remained true even if the hs-CRP level remained within the normal range. hs-CRP can be used as a predictor of NAFLD, as well as other obesity-associated diseases. Therefore, individuals with higher hs-CRP levels (even within the normal range) may require appropriate follow-up and management to prevent NAFLD development.

## Introduction

Non-alcoholic fatty liver disease (NAFLD) is characterized by triglyceride accumulation in hepatocytes, which occurs without alcohol abuse. It is one of the most commonly encountered chronic liver diseases [1]. The clinical spectrum of NAFLD ranges from asymptomatic steatosis to steatohepatitis, fibrosis and cirrhosis. Most NAFLD patients have asymptomatic simple steatosis without adverse sequelae [2]. Only 3–5% of NAFLD patients suffer from the active form of NAFLD, known as steatohepatitis (NASH) [3]. Patients with NASH may develop liver fibrosis, and have an increased risk of liver cirrhosis and hepatocellular carcinoma, resulting in end-stage liver disease [2–6].

NAFLD is the most common cause of abnormal liver function tests in the United States, with a prevalence of 14–24% of the general population [6,7]. Approximately 15–30% of the Asian population is reported to have NAFLD. In addition over 50% of those with diabetes and metabolic syndrome in Asia have NAFLD[5]. In South Korea, the reported prevalence of NAFLD is 15–30%, and growing due to the aging population and increased prevalence of obesity and diabetes[3]. NAFLD is recognized as a main cause of chronic liver disease, not only in Western countries, but also in Asia[3]. Given its associations to obesity, hypertension, dyslipidemia, and insulin resistance, NAFLD is traditionally considered a hepatic component of metabolic syndrome(MetS) [8]. Recently, studies have reported that NAFLD is an independent risk factor of numerous diseases, including diabetes, cardiovascular disease, hypertension, kidney disease and colon cancer. Therefore, interest is growing with regard to NAFLD [8–11].

Low-grade inflammation is known to play a role in MetS, cardiovascular disease, type 2 diabetes, hypertension, some cancers, as well as NAFLD [12]. An elevation in hs-CRP, a marker of chronic inflammation, can predict various obesity related diseases [13–18]. Since NAFLD is considered a hepatic component of metabolic syndrome, it can also be predicted by elevated hs-CRP levels [19–23].

However, most prior studies found that NAFLD is only correlated with hs-CRP levels that are elevated beyond the normal range, not those within the reference interval [21,22]. Furthermore, most NAFLD diagnoses in these studies was made using ultrasonography (US), making it difficult to collect mass data; therefore, most prior studies were relatively small. In addition, most of these studies were cross-sectional [19,20,22]. Therefore, they were not able to demonstrate causality between hs-CRP level and NAFLD.

We performed this retrospective cohort study in healthy Korean men to determine whether higher hs-CRP levels (within the normal range) could predict NAFLD development. Furthermore, we investigated whether following the hs-CRP level, even if normal, could be beneficial in the prevention of NAFLD.

## Materials and methods

### Study population

We conducted a retrospective cohort study of Korean male workers at one of the largest semiconductor manufacturing companies and its 13 affiliates [24–26]. According to the Korean Industrial Safety and Health Law, all employees are obliged to participate in either annual or biennial health check-ups. We collected the results of these health examinations of men aged 30 to 59 years old between January 2002 and December 2002. These results created a baseline study population. Among 15,347 workers who had health examinations at a university hospital in Seoul, Korea, we excluded 824 men without hs-CRP levels. We also excluded 9928 men based on the following criteria at baseline (some individuals met more than one exclusion criterion): (a) 5052 men with fatty liver on ultrasound; (b) 4132 men with abnormal hs-CRP levels; (c) 1292 had increased Alanine aminotransferase (ALT)(≥35U/L); (d)438 had increased homeostasis model assessment-insulin resistance(HOMA-IR) [27]; (e) 1437 reported an alcohol intake≥20g/day; (f) 376 had a positive serologic finding for hepatitis B or C virus; (g) 438 had metabolic syndrome (MetS) [8]; (h)17 had a history of malignancy; (i)11 had a history of cardiovascular disease; (j)169 had hepatitis or liver cirrhosis; (k)38 were under treatment for dyslipidemia; (l)59 had diabetes; (m)28 were taking medications that might affect hs-CRP level.

**Fig 1:**
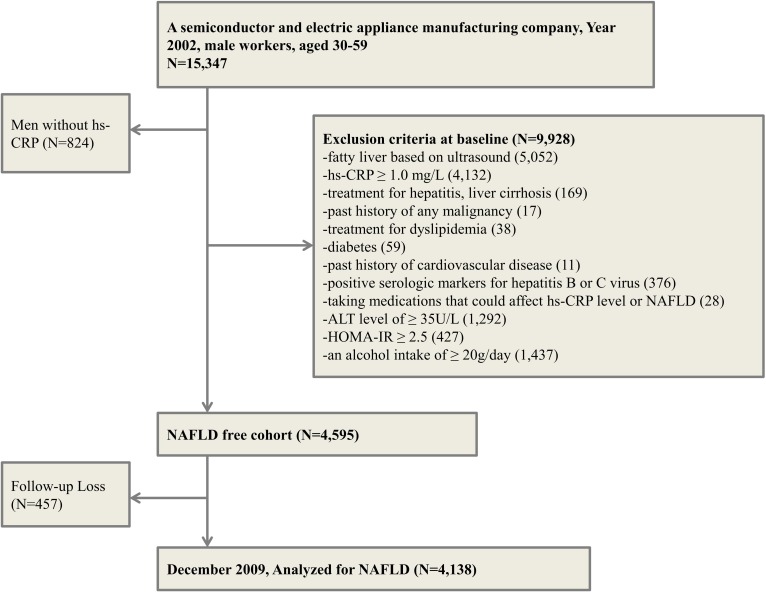
Flow diagram of study subject selection.

The NAFLD-free cohort was comprised of 4,595 men. These participants were reexamined at the same hospital annually or biennially through December 2009. After excluding 457 subjects who did make at least one follow-up examination, 4138 men were finally analyzed (Fig 1). The follow-up person-year was 54,663. This study was approved by the Institutional Review Board at Kangbuk Samsung Hospital. The informed consent requirement for this study was exempted by the Institutional Review Board because at the time of health examination the study was in the planning phase. Researchers accessed the database for analysis purposes only, which was free of identifying personal information.

### Measurements

Laboratory procedures have previously been documented in detail [24–26]. Questions regarding alcohol consumption entailed the frequency of drinking on a weekly basis and the usual amount consumed each day to calculate the average amount of alcohol intake per week, and current smoking was recorded. Participants who reported moderate- or vigorous- physical activity (such as jogging, bicycling, and swimming) more than once per week were considered regular exercisers.

Venous blood was collected from an antecubital vein after at least 12h of fasting. The Bayer Reagent Packs on an automated chemistry analyzer (Advia 1650™ Autoanalyzer; Bayer Diagnostics, Leverkusen, Germany) was used to measure serum levels of FBG, total cholesterol (TC), triglycerides (TG), low-density lipoprotein-cholesterol (LDL-C), high-density lipoprotein-cholesterol (HDL-C), γ-glutamyltransferase (GGT), ALT, aspartate aminotransferase (AST), and alkaline phosphatase levels. The hexokinase method was used to measure glucose; the enzymatic colorimetric tests were used to measure total cholesterol and serum triglycerides; the homogeneous enzymatic colorimetric test for LDL cholesterol; the selective inhibition method (Bayer Diagnostics) for HDL cholesterol; and immunoradiometric assays (Biosource) for insulin concentrations.

Insulin resistance was calculated as (fasting glucose × fasting insulin)/22.5, according to the homeostasis model assessment of insulin resistance (HOMA-IR) [27]. We analyzed high sensitivity-C reactive protein (hs-CRP) using particle-enhanced immunonephelometry with the BNII™ System (Dade Behring, Marburg, Germany) and a lower detection limit of 0.175 mg/L. hs-CRP was calculated by rounding off the numbers to two decimal places. For quality control, the laboratory undergoes annual inspections and surveys by the Korean Association of Quality Assurance for Clinical Laboratories.

Sitting blood pressures were measured by trained nurses using a standard mercury sphygmomanometer. Height and weight were measured to the nearest 0.1 kilograms and 0.1 centimeters respectively with study participants wearing light hospital gown and no shoes. The body mass index (BMI) was calculated as the patient’s weight (in kilograms) divided by the square of the patient’s height (in meters).

### Definition

Fatty liver was determined based on the results of abdominal ultrasonography with a 3.5-MHz transducer(Logic Q700 MR; GE, Milwaukee, Wisconsin, USA) [24]. All hepatic ultrasounds were captured and read by board certified radiologists, who were not aware of the aims of the study and were blinded to laboratory values. Images were taken in a standard fashion with the study subjects in the supine position with the right arm raised above the head [24]. Among the four known criteria for the diagnosis of fatty liver (including hepatorenal echo contrast, liver brightness, deep attenuation, and vascular blurring) [28], participants with hepatorenal contrast and liver brightness are considered to have fatty liver [24,29]. Based on computer-generated random samples among the stored images, there was excellent agreement on fatty liver diagnosis between the three radiologists (agreement 99%, κ = 0.98).

Metabolic syndrome was defined as three or more of the following criteria, as proposed by the ATP III[30]: (a) abdominal obesity; (b) high fasting glucose: ≥6.1 mmol/L; (c) hypertriglyceridemia: triglycerides ≥1.69 mmol/L; (d) low HDL cholesterol: ≥1.04 mmol/L; and (e) high blood pressure: ≥130/85 mmHg. Since waist measurements were unavailable for all study participants, we substituted abdominal obesity for the cutoff value of BMI≥25 kg/m^2^, as proposed for the diagnosis of obesity in Asian people [31,32].

### Statistical analysis

As described, we retrospectively analyzed the health examination data of 4,138 participants from January 2002 to December 2009. We only included men with normal baseline hs-CRP levels. Therefore, the distribution of baseline hs-CRP levels was quite narrow. We divided the study population into three groups according to their baseline hs-CRP levels: 0–0.2, 0.3–0.4, and 0.5–0.9mg/L. Since the lowest hs-CRP level was reported as “below 0.2mg/L”, the first group (range 0–0.2mg/L) was comprised of 1750 men (42.3%), the second group 1168 (28.2%), and the third group 1220 (29.5%).We used the χ^2^-test and 1-way ANOVA to analyze statistical differences according to the characteristics of the study participants in 2002 with regard to a normal-range hs-CRP level. If the variable did not show homoscedasticity, a robust ANOVA (Welch test) was performed. The Bonferroni comparison test (if, if robust ANOVA, post-hoc test is Tamhane’s test) was used to make comparisons between the three groups.

For incident NAFLD cases, the time of disease onset was assumed to be the midpoint between the visit at which NAFLD was diagnosed and the previous visit. Once NAFLD was diagnosed, the case was included as an “event,” and follow-up period was terminated. Cases without NAFLD onset were considered to be “censored.”

We used the Cox proportional hazard model to determine the relationship between the three hs-CRP groups and NAFLD development. The incidence rate was expressed as the number of cases divided by 1000 person-years from baseline until NAFLD development. We estimated the hazard ratios using 95% CI in three models: non-adjusted, age-adjusted, and multivariable adjusted. In the multivariate models, we included age, smoking, exercise, BMI, triglyceride, SBP and fasting serum glucose.

Statistical data analyses were performed using IBM SPSS version 19.0 (SPSS Inc., Chicago, IL, USA). P-values <0.05 were considered statistically significant.

## Results

The baseline characteristics of the study subjects are summarized in Table 1. The mean (SD) age of the 4138 men was 36.5 years (4.7). The variables related to MetS, including BMI, SBP, DBP, fasting serum glucose and triglyceride, were all significantly correlated with increasing hs-CRP. In contrast, the HDL cholesterol level was negatively correlated with increasing hs-CRP, as expected. BMI and HDL cholesterol were significantly different across all three groups of hs-CRP. Uric acid, insulin, and HOMA-IR also demonstrated a linear trend in relation to hs-CRP groups. Hepatic enzymes, such as AST, ALT, and GGT, were significantly increased in a dose-response manner in relation to the hs-CRP groups. Of all participants, 43.3% were current smokers and 50.3% were regular exercisers. Participants in the higher hs-CRP group were more likely to be regular exerciser than those in the lower group. In contrast, there was no association between group and smoking frequency.

**Table 1:** Baseline participant characteristics by hs-CRP category.

	Overall	hs-CRP categories			p-value	Multiple comparison
		I	II	III		
Number	4138	1750 (42.3)	1168 (28.2)	1220 (29.5)		
Range (mg/L)		0 to 0.2	0.3 to 0.4	0.5 to 0.9		
Age (years) *	36.5 (4.7)	36.1 (4.6)	36.7 (4.8)	36.8 (4.6)	<0.001	I≠II, III
BMI (kg/m^2^) *	22.5 (2.2)	21.9 (2.2)	22.7 (2.2)	23.2 (2.2)	<0.001	I≠II, III & II≠III
Systolic BP (mmHG) *	113.2 (11.5)	112.7 (11.4)	113.0 (11.1)	114.0 (11.8)	0.008	I≠III
Diastolic BP (mmHG) *	73.2 (9.2)	72.7 (9.1)	73.4 (8.8)	73.8 (9.5)	0.002	I≠III
Glucose (mg/dl) *	89.3 (9.0)	88.8 (8.3)	89.4 (9.7)	89.9 (9.4)	0.004	I≠III
Total cholesterol (mg/dl) *	193.9 (31.6)	190.4 (31.5)	195.5 (30.6)	197.3 (32.0)	<0.001	I≠II, III
HDL-C (mg/dl) *	54.7 (11.6)	56.3 (12.1)	54.6 (11.3)	52.6 (10.8)	<0.001	I≠II, III & II≠III
LDL-C (mg/dl) *	115.8 (27.2)	112.7 (27.2)	117.1 (26.5)	118.9 (27.4)	<0.001	I≠II, III
Triglyceride (mg/dl) *	118.9 (60.7)	110.0 (52.3)	122.2 (62.2)	128.5 (68.2)	<0.001	I≠II, III
Uric acid (mg/dl) *	5.78 (1.04)	5.69 (0.99)	5.82 (1.06)	5.89 (1.08)	<0.001	I≠II, III
AST (U/L)†	21 (19–24)	21 (18–23)	21 (19–24)	22 (19–24)	<0.001	I≠II, III & II≠III
ALT (U/L)†	20 (16–24)	19 (15–23)	20 (16–25)	21 (17–25)	<0.001	I≠II, III & II≠III
GGT (U/L)†	19 (14–25)	17 (14–32)	19 (15–25)	21 (16–28)	<0.001	I≠II, III & II≠III
Insulin (μIU/ml)†	6.11 (4.99–7.66)	5.91 (4.87–7.37)	6.13 (4.99–7.75)	6.38 (5.17–8.01)	<0.001	I≠II, III & II≠III
HOMA-IR†	1.33 (1.07–1.71)	1.27 (1.04–1.64)	1.34 (1.06–1.73)	1.41 (1.13–1.81)	<0.001	I≠II, III & II≠III
Current smoker (n,%)	1773 (43.3)	792 (45.9)	466 (40.3)	515 (42.6)	0.013	
Regular exerciser (n,%)§	2063 (50.3)	817 (47.1)	575 (49.7)	671 (55.3)	0.001	

Data are *the means (standard deviation), †medians (interquartile range), or percentages. §>1 time/week. hs-CRP, high sensitivity C-reactive protein; ALT, alanine aminotransferase; AST, aspartate aminotransferase; BMI, body mass index; BP, blood pressure; GGT, gamma-glutamyltranspeptidase; HDL-C, high-density lipoproteincholesterol; HOMA-IR, homeostasis model assessment of insulin resistance; LDL-C, low-density lipoprotein-cholesterol. p-value; ANOVA or chi-square test (robust ANOVA; Welch test). Multiple comparison test; Bonferroni test (if robust ANOVA, post-hoc test is Tamhane’s test).

**Fig 2:**
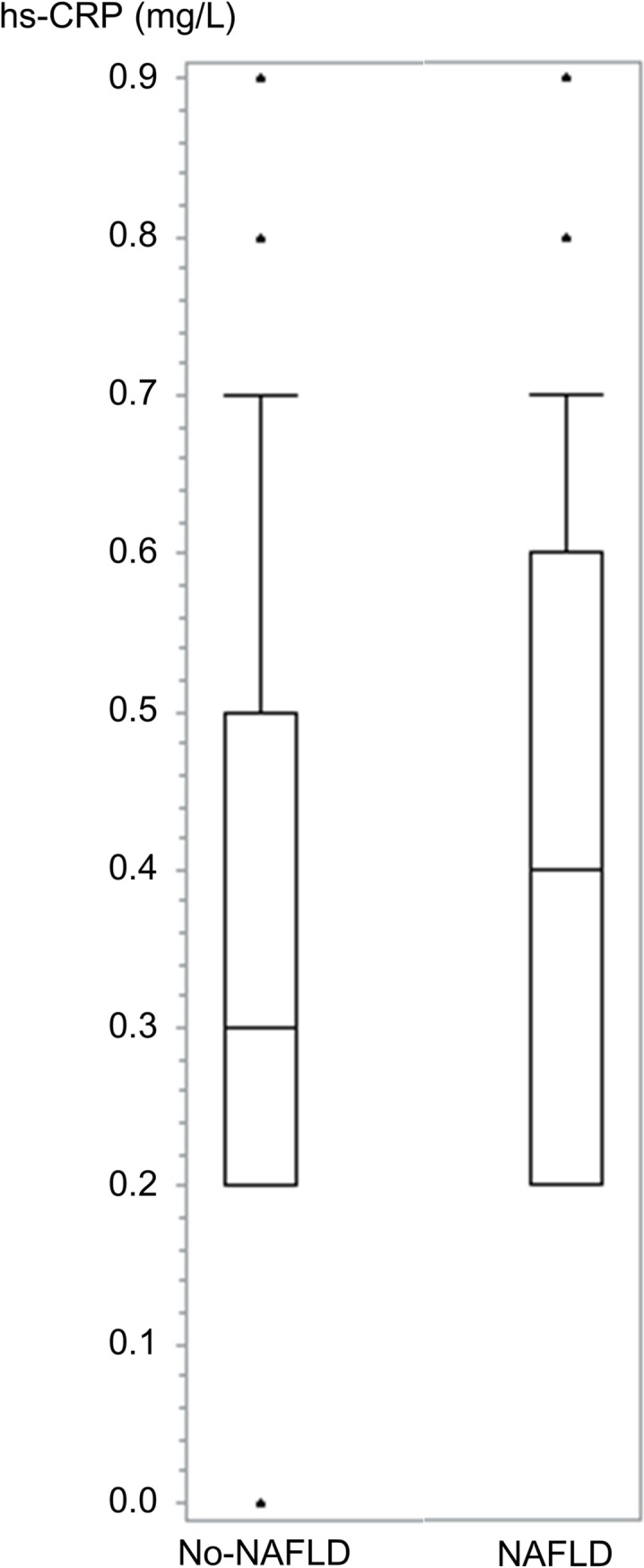
Distribution of hs-CRP between NAFLD and no-NAFLD.

The major risk factors for the development of NAFLD are demonstrated in Table 2. Age, BMI, SBP, fasting serum glucose and triglyceride are significantly higher in subjects who developed NAFLD than those who didn’t developed NAFLD. The percentages of current smokers and regular exerciser were higher in NAFLD group, however only current smoker was statistically significant. Numbers and percentages of hs-CRP groups for the development of NAFLD are also shown. The highest hs-CRP group was 25.8% in participants without NAFLD, 38.6% in participants with NAFLD, respectively. We also found the difference of hs-CRP distribution between NAFLD and no-NAFLD (Fig 2).

**Table 2:** Baseline participant characteristics between NAFLD and no NAFLD.

	No NAFLD	NAFLD	p-value
n	2947	1191	
Age (years) *	36.4 (4.7)	36.6 (4.6)	0.276
BMI (kg/m^2^) *	22.2 (2.2)	23.4 (2.0)	<0.001
Systolic BP (mmHg) *	112.9 (11.4)	113.9 (11.6)	0.016
Glucose (mg/dl) *	89.0 (9.4)	90.0 (8.2)	0.001
Triglyceride (mg/dl) *	109.9 (52.7)	141.0 (72.4)	<0.001
Current smoker (n,%)	1225 (42.1)	548 (46.5)	0.010
Regular exerciser (n,%)§	1454 (49.8)	609 (51.3)	0.391
hs-CRP			
0.2	1344 (45.6)	406 (34.1)	<0.001
0.3–0.4	843 (28.6)	325 (27.3)	<0.001
0.5–0.9	760 (25.8)	460 (38.6)	<0.001

Data are *the means (standard deviation) or percentages. §>1 time/week. BMI, body mass index; BP, blood pressure; hs-CRP, high sensitivity C-reactive protein.

The risk of developing NAFLD in relation to hs-CRP group is shown in Table 3. The incident rate, hazard ratios, 95% CI and p-value for trends according to hs-CRP group are specified in the following three models: non-adjusted, age-adjusted, and multivariable-adjusted. During follow up, 1191 participants developed NAFLD; 406 in the lowest hs-CRP group, 325 in the middle group and 460 in the highest group. The HRs of NAFLD increased according to the hs-CRP groups in a dose-responsive manner. The HR values (95% CI) from the middle hs-CRP group and the highest hs-CRP group were 1.24(1.07–1.44) and 1.76(1.54–2.01), respectively. In the age-adjusted model, the HR values (95% CI) from the middle hs-CRP group and the highest hs-CRP group still increased significantly at 1.23(1.07–1.43) and 1.74(1.52–1.99), respectively. After adjusting for age, exercise, smoking, BMI, triglyceride, SBP, fasting serum glucose, significantly elevated HRs were only found between the lowest hs-CRP group and the highest hs-CRP group. The p-value for the trend of HR values of NAFLD was<0.001 in all three models.

**Table 3:** Hazard ratios (95% CI) for the incidental rate of non-alcoholic fatty liver by the baseline hs-CRP level (Cox proportional hazard models).

CRP (mg/L) category	Incident case (n)	Person-years	Incidental rate (n/1,000 person-years)	HR (95% CI)	Age-adjusted HR (95% CI)	Multivariable-adjusted HR(95% CI) *
0.2	406	19018.2	21.35	1.00	1.00	1.00
0.3–0.4	325	18335.5	17.73	1.24 (1.07–1.44)	1.23 (1.07–1.43)	1.01 (0.87–1.18)
0.5–0.9	460	17309.3	26.58	1.76 (1.54–2.01)	1.74 (1.52–1.99)	1.28 (1.12–1.48)
P for trend				< 0.001	< 0.001	< 0.001

*Estimated from Cox proportional hazard models, adjusted for age, smoking, exercise, BMI, triglyceride level, SBP, and fasting serum glucose. hs-CRP, high sensitivity C-reactive protein; BMI, body mass index; CI, confidence intervals; HR, hazard ratios.

## Discussion

To our knowledge, this is the first retrospective cohort study to determine whether normal-range hs-CRP values can predict NAFLD development. Using the Cox proportional hazard model, we found that healthy men with no metabolic abnormalities, but with relatively high hs-CRP levels (still within the normal range) had a higher risk of developing NAFLD than did those with lower-range hs-CRP levels. This association was still significant after adjusting for age, exercise, smoking, BMI, triglyceride, SBP, and fasting serum glucose. Since NAFLD is considered a hepatic manifestation of the metabolic syndrome [8,11], the association between NAFLD and low-grade inflammation has been reported in many studies [19–23]. The hs-CRP level is known to be an independent predictor of cardiovascular disease, as well as metabolic abnormalities, since low-grade inflammation plays a major role in the pathogenesis [33–35].

In a case-control study on Asian Indians, NAFLD was independently related to sub-clinical inflammation [23]. Chian et al. found that increasing hs-CRP level not only correlates with NAFLD severity, but also cardiovascular risk [19]. In another case-control study, inflammatory markers such as TNF-α, IL-6 and hs-CRP levels were higher in the NAFLD group than in healthy controls [20]. In one study by Kappan et al., inflammatory markers were higher in subjects with NAFLD than they were in those without; this finding was still significant after adjusting for metabolic components, suggesting the pathologic role of low grade inflammation on NAFLD development [21]. However, most studies were cross-sectional or case-control, preventing the conclusion of any causal relationships [19,20,22]. In addition, most previous studies focused on the relationship between higher hs-CRP level and NAFLD, rather than a normal-range hs-CRP level and NAFLD [21,22]. Using a retrospective cohort model, we followed healthy men for 7 years to determine whether a relatively higher level of hs-CRP within the normal range increases the risk of NAFLD development.

The pathogenesis of NAFLD is not fully understood. It is clear that hepatic steatosis is more frequent with obesity and MetS, both of which are known pro-inflammatory conditions [22]. Insulin resistance and oxidative stress are considered to be two key mechanisms of NAFLD. Both are associated with the hs-CRP level [36]. The macrophages in adipose cells secrete proinflammatory cytokines (adipocytokines) such as C-reactive protein, interleukin (IL)–6, IL-8, IL-10, and tumor necrosis factor–α; these cytokines impair insulin signaling, inducing insulin resistance [20,37]. Insulin resistance also promotes hepatic lipid accumulation by increasing fatty acids in the liver[38]. The correlations between hs-CRP level and total cholesterol, HDL-cholesterol, triglyceride, and insulin levels in this study can be explained by the role of cytokines in insulin resistance and lipid accumulation. The second mechanism, oxidative stress, acts independently from cytokine-driven intra-hepatic inflammation. Triglyceride accumulation in the liver increases oxidative stress, which induces more inflammation and results in liver injury [6,36]. Patients with reduced antioxidant capacity had higher levels of hs-CRP [39]. Patients with NAFLD are also reported to have low antioxidant capacity [36,40]. Abdominal obesity and metabolic syndrome predispose to hepatic steatosis. Both of these risk factors do so through the increased delivery of free fatty acids to the liver and increases in hepatic lipogenesis associated with hyperinsulinemia[6]. However, NAFLD is found in persons with and without obesity. Our finding that relatively higher hs-CRP levels increases the risk of NAFLD, even after adjusting for metabolic factors, can be explained by chronic low-grade inflammation through these two mechanisms. This result support Yatsuzuka et al. [41] that hs-CRP is associated with fatty liver, independent of visceral fat volume and can be used as a diagnostic marker of fatty liver. It also corresponds to other studies that found that fatty liver, obesity, and metabolic syndrome are independently and additively related to systemic inflammation [22].

Our study has several limitations. First, fatty liver was diagnosed using ultrasound. Although liver biopsy is the gold standard for hepatic steatosis identification, it is too invasive to be conducted on healthy populations during routine health check-ups. Ultrasound is a practical tool that is commonly used in other studies regarding fatty liver [21–23]. In addition, since the radiologists who conducted the ultrasounds were not aware of the study’s aim, information bias was likely avoided. A second limitation is that the study population (of 30–59 year old Korean men) may prevent its generalization to other populations. This study was also conducted on healthy men only so it is difficult to generalize on women. Thirdly, the sensitivity and specificity of hs-CRP for the prediction to NAFLD was not acceptable (Sensitivity 0.66 and Specificity 0.38 if cut-off was set as 0.3). So, we authors thought that we could only suggest the association between hepatic steatosis and low level of systemic inflammation. In the current situation, we thought that it is not possible to make a new cut-off value.

Despite these limitations, we were able to assess a causal relationship between hs-CRP and NAFLD over a 7 year-follow-up of a large population. There are few studies that analyze a large number of abdominal ultrasonographic data for NAFLD. This study also alludes to the hazard ratio and potential mechanisms explaining how healthy men without metabolic abnormalities develop NAFLD.

## Conclusion

This is the first study to document that higher levels of hs-CRP, even within the normal limits, increase one’s risk of NAFLD. Our findings support the concept of an independent association between hepatic steatosis and systemic inflammation. A relative elevation in hs-CRP among patients with hepatic steatosis may serve as a marker of NAFLD development, as well as of MetS, cardiovascular disease, diabetes, hypertension and some cancers. Therefore, healthy individuals with high-normal hs-CRP levels should be followed closely for potential NAFLD development and its associated comorbidities.

## Supporting information

S1 Dataset Raw dataset of the study. (ZIP)
