# Correction: High-normal levels of hs-CRP predict the development of non-alcoholic fatty liver in healthy men

**DOI:** 10.1371/journal.pone.0206834

**Published:** 2018-10-29

**Authors:** Jieun Lee, Kijung Yoon, Seungho Ryu, Yoosoo Chang, Hyoung-Ryoul Kim

The affiliation for the second author is incorrect. Kijung Yoon is not affiliated with #2 but with: Independent researcher, Ridgewood, New Jersey, United States.
